# Bacterial lipoprotein plays an important role in the macrophage autophagy and apoptosis induced by *Salmonella typhimurium* and *Staphylococcus aureus*

**DOI:** 10.1515/biol-2022-0739

**Published:** 2023-09-30

**Authors:** Shanshan Jiang, Jinyao He, Lijie Zhang, Qiaojiajie Zhao, Shuqi Zhao

**Affiliations:** Institute of Hematological Research, Shaanxi Provincial People’s Hospital, Xi’an, Shaanxi, 71000, China; Clinical Laboratory, Xi’an Medical University, Xi’an, Shaanxi, 710068, China

**Keywords:** bacterial lipoprotein, autophagy, apoptosis, bone marrow-derived macrophages, bacterial infection

## Abstract

This study aimed to determine the role of bacterial lipoprotein (BLP) in autophagy and apoptosis. Western blot was used to examine autophagy biomarkers in mouse bone marrow-derived macrophages (BMDMs) after infection with *Salmonella typhimurium* (*S. typhimurium*) and *Staphylococcus aureus* (*S. aureus*) and BLP stimulation. In BMDMs, enhanced protein expression of LC3-II was observed after *S. typhimurium* or *S. aureus* infection (*P* < 0.05) and BLP stimulation (*P* < 0.05). Autophagy inhibition by chloroquine resulted in increased levels of LC3-Ⅱ and p62 protein (*P* < 0.05). Persistently upregulated expressions of Atg3 and Atg7 were observed following BLP stimulation (*P* < 0.05), and knockdown of Atg3 or Atg7 significantly attenuated BLP-enhanced protein expression of LC3-Ⅱ in BMDMs. Furthermore, we found that the autophagy inhibitor 3-methyladenine prevented BLP- and infection-induced macrophage apoptosis. BLP is not only required for autophagy and apoptosis activation in macrophages but also for regulating the balance between autophagy and apoptosis.

## Graphical abstract

BLP as a bacteria component is an important factor in autophagy activation and induces apoptosis. BLP-induced macrophage apoptosis was suppressed by inhibiting autophagy.

**Figure:**
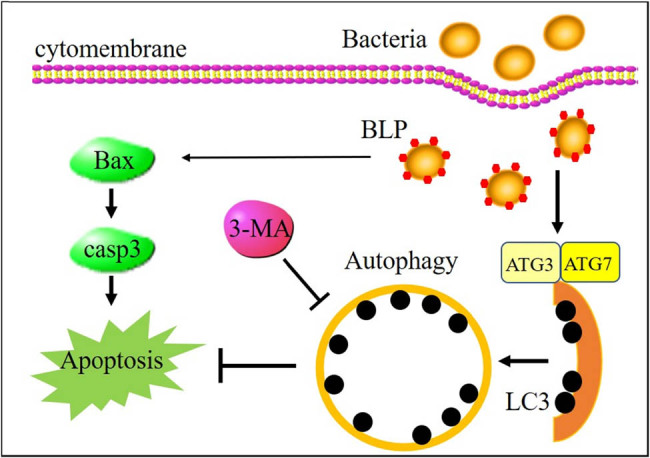

## 1 Introduction

Sepsis and related diseases are caused by the activation of an inflammatory cascade triggered by bacterial components such as the endotoxin lipopolysaccharide (LPS), exotoxin, and cell wall components [1]. Macrophages recognize bacterial components and activate the innate immune response, which targets and destroys bacterial pathogens via a vast array of receptors, immune signaling pathways, and cellular processes, including Toll-like receptors (TLRs), Nod-like receptors, phagocytosis, autophagy and apoptosis [2,3]. Antimicrobial autophagy clears or limits the spread of infection by capturing and delivering pathogens to lysosomes [4,5]. However, if autophagy is unable to prevent severe and persistent infections, cells can activate apoptosis to ensure self-elimination and avoid local inflammation and pathogen spread [6]. Although the biochemical and morphological characteristics of autophagy and apoptosis are fundamentally different, the regulatory and executing protein networks are highly interconnected.

Previous studies revealed that LPS, a major toxin in the outer membrane of Gram-negative bacteria, causes sepsis and promotes inflammation by TLR4 and its downstream signaling pathways. LPS also induces autophagy and apoptosis in macrophages [7]. Bacterial lipoprotein (BLP, Pam3CSK4), TLR2 agonist, is another important surface component of both Gram-negative and -positive bacteria that contributes not only to their function but also to pathogenesis such as virulence, colonization, and evasion from immune responses [8,9]. Furthermore, BLP, like LPS, causes inflammation in macrophages. For example, BLP activates macrophages via TLR2 and the downstream cascade, which is initiated by an interaction between the Toll/interleukin-1 receptor and myeloid differentiation primary response protein 88. However, whether BLP induces autophagy and apoptosis needs to be further investigated [7,10].

In the present study, macrophages were treated with different bacteria to establish an *in vitro* infection model or stimulated with BLP. Our data show that BLP can cause autophagy and apoptosis on its own. Hence, bacteria activate autophagy and induce apoptosis of macrophages by BLP.

## 2 Materials and methods

### 2.1 Reagents and antibodies

BLP (Pam3Cys-Ser-Lys4, ab142085) was purchased from Abcam (Cambridge, UK). Gram-negative *S. typhimurium* (CMCC50097) and Gram-positive *S. aureus* (ATCC6538) were obtained from the Laboratory of Pathogenic Microorganism, Southern Medical University, Guangzhou, China. Antibodies against autophagy-related proteins (Autophagy Antibody Sampler kit #4445) and chloroquine (CQ) were purchased from Cell Signaling Technology (Beverly, MA, USA). Antibodies against Bcl2 (#26593-1-AP), Bax (#50599-2-Ig), and Caspase3 (#19677-1-AP) were obtained from ProteinTech (Chicago, IL, USA). We purchased 3-methyladenine (3-MA) (#189490) from Sigma-Aldrich (St. Louis, MO, USA). The siRNA targeting Atg3, Atg7, and scrRNA were obtained from GenePharma (Shanghai, China).

### 2.2 Cell and bacterial cultures

Male C57BL/6 mice (6–8 weeks) were obtained and maintained in the animal facility of Southern Medical University, Guangzhou, China. The isolation and culture of bone marrow-derived macrophages (BMDMs) from mice were conducted as described previously [11]. Briefly, mice were euthanized by cervical dislocation under anesthesia, and the isolated femur and tibia bones were flushed with DMEM to obtain marrow. Samples were resuspended in DMEM supplemented with 20% L929 conditioned medium, 20% fetal bovine serum, 100 U/ml penicillin, and 100 mg/ml streptomycin sulfate and cultured for 7 days [12]. The protocol was approved by the Committee on Animal Research and Ethics.

Bacteria were cultured in Luria–Bertani broth (Sigma-Aldrich, St. Louis, MO, USA) at 37°C and resuspended in DPBS (Invitrogen Life Technologies, Paisley, Scotland, UK) when they reached the mid-logarithmic growth phase. The concentration of resuspended bacteria was determined by generating serial 10-fold dilutions and then plating and counting the bacterial CFU. To heat-kill bacteria, bacteria were incubated at 100°C for 30 min.


**Ethical approval:** The research related to animal use complied with all the relevant national regulations and institutional policies for the care and use of animals, and has been approved by the Committee on Animal Research and Ethics of Southern Medical University (SCXK 2016-0041).

### 2.3 RNA interference

BLP- and mock-treated BMDMs were transfected with Atg3 or Atg7-specific siRNA or scrambled siRNA (scrRNA) using a NEPA21 super electroporator (Nepagene, Chiba, Japan). Total RNA was extracted 48 h post-transfection and analyzed by Western blotting to determine the interference efficiency.

### 2.4 Western blotting

BMDMs (2 × 10^6^/dish) were incubated with bacteria at a ratio of 1:30 at 37°C for various time periods. Cells were lysed, and equal protein amounts of lysates were separated on 12.5% SDS-polyacrylamide gels before being transferred to PVDF membranes, which were then blocked at room temperature with PBS containing 0.05% Tween-20 and 5% nonfat milk for 2 h. Membranes were then incubated with primary antibodies overnight at 4°C. After 1 h of probing with anti-rabbit secondary antibodies at room temperature, the PVDF membranes were developed with a chemiluminescent substrate and visualized on a Kodak IS4000R (Kodak, NY, USA). Densitometric analysis was performed with ImageJ software, version 1.42 (National Institutes of Health, Bethesda, MA, USA).

### 2.5 Annexin V-FITC/PI double staining for apoptosis detection

BMDMs (2 × 10^5^/dish) were incubated with bacteria at a ratio of 1:30 at 37°C for various time periods. At the end of the treatment, apoptosis was measured using an annexin Ⅴ-FITC/PI apoptosis assay kit (Beyotime Biotechnology, Shanghai, China). In brief, BMDMs were dissociated and centrifuged before being resuspended in 100 μl of binding buffer with 5 μl of annexin V-FITC and 10 μl of PI. After incubation for 20 min at room temperature in the dark, the sample was immediately analyzed by the BD FACSVerse^TM^ flow cytometer. The Flow Cytometry Standard files were analyzed using FlowJo software8.0.1.

### 2.6 Statistical analysis

SPSS 17.0 (IBM Corp, USA) was used to analyze data, which were presented as mean ± standard deviation (SD). A *T*-test was used to compare the two groups. One-way ANOVA was used to compare multiple groups. All experiments were repeated at least three times, and *P* < 0.05 was set to indicate statistical significance.

## 3 Results

### 3.1 *S. typhimurium* and *S. aureus* induce autophagy in macrophages

**Figure 1:**
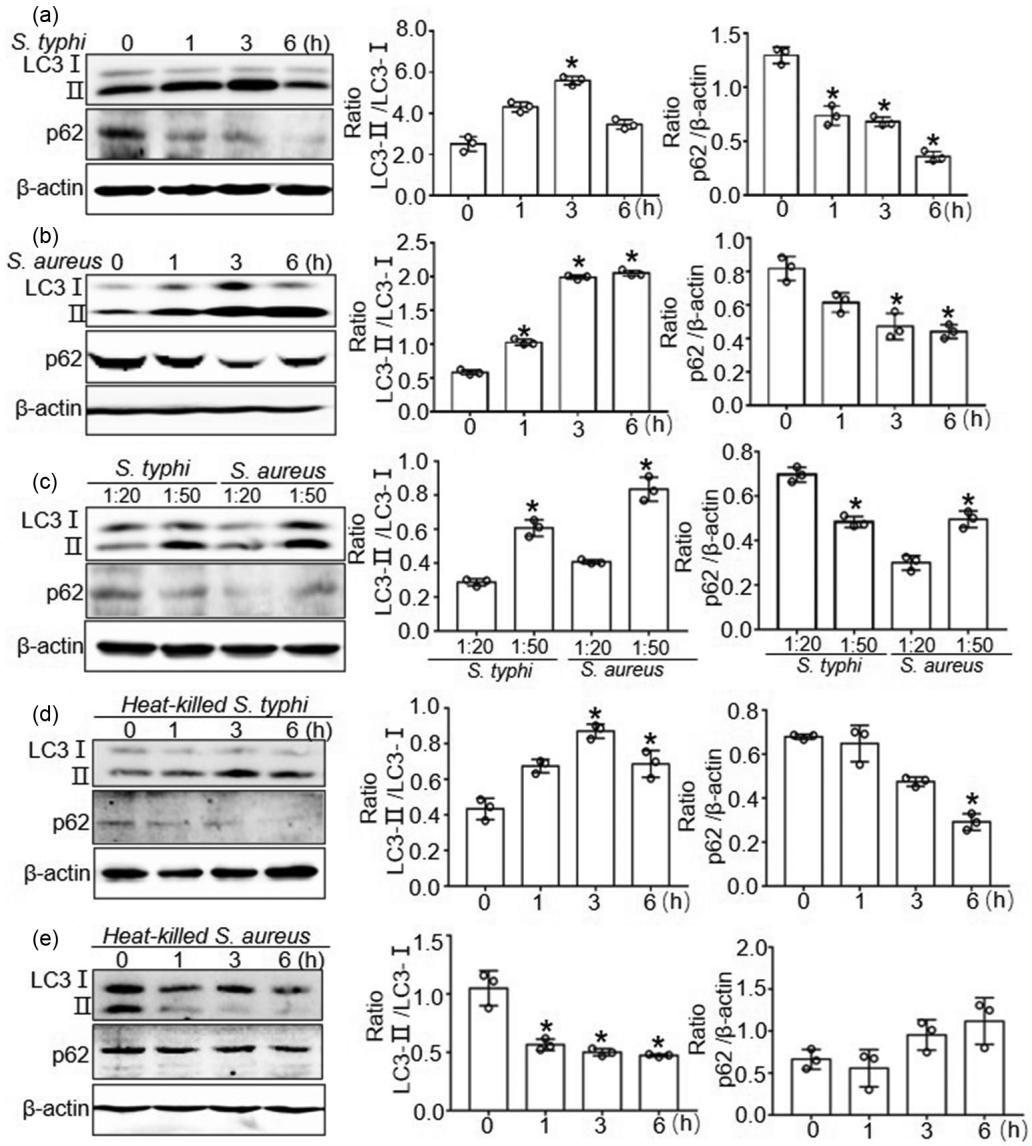
Enhanced autophagy in macrophages upon *S. typhimurium* or *S. aureus*. (a) and (b) The protein expression of LC3 and p62 in BMDMs at 0, 1, 3, 6 h after *S. typhimurium* or *S. aureus* infection. Western blot was quantitatively analyzed, and data shown are mean ± SD from three independent experiments. *P < 0.05 compared with 0 h. (c) The protein expression of LC3 and p62 in BMDMs after different numbers of *S. typhimurium* or *S. aureus* infection. Western blot was quantitatively analyzed, and data shown are mean ± SD from three independent experiments. *P < 0.05 compared with different bacterial number. (d) and (e) The protein expression of LC3 and p62 in BMDMs at 0, 1, 3, and 6 h after heat-killed *S. typhimurium* or *S. aureus* infection. Western blot was quantitatively analyzed, and data shown are mean ± SD from three independent experiments. *P < 0.05 compared with 0 h.

To investigate whether *S. typhimurium* and *S. aureus* induce autophagy in BMDMs, the expression levels of autophagy biomarkers such as LC3 and p62 were examined by Western blot. Enhanced protein expression of LC3-Ⅱ was observed at 1 and 3 h after *S. typhimurium* infection and at 1, 3, and 6 h after *S. aureus* stimulation in BMDMs compared with the expression at 0 h (*P* < 0.05) (Figure 1a and b). Next, we tested the effect of different bacterial quantities on autophagy. The results showed that a higher quantity of bacteria led to increased induction of autophagy (Figure 1c).

To investigate the mechanism of bacteria-induced autophagy, we examined LC3 and p62 expressions after heat-killed *S. typhimurium* and *S. aureus* infection at 1, 3, and 6 h. The protein expression of LC3-Ⅱ still increased after the administration of heat-killed *S. typhimurium.* However, the protein expression of LC3-Ⅱ decreased while the p62 level was unchanged after infection by heat-killed *S. aureus* (Figure 1d and e). These findings indicate that live *S. aureus* infection enhances autophagy in macrophages, but heat-killed *S. aureus* did not have this effect. However, both live and heat-killed *S. typhimurium* infection induces autophagy.

### 3.2 *S. typhimurium* and *S. aureus* accelerate autophagic flux

**Figure 2:**
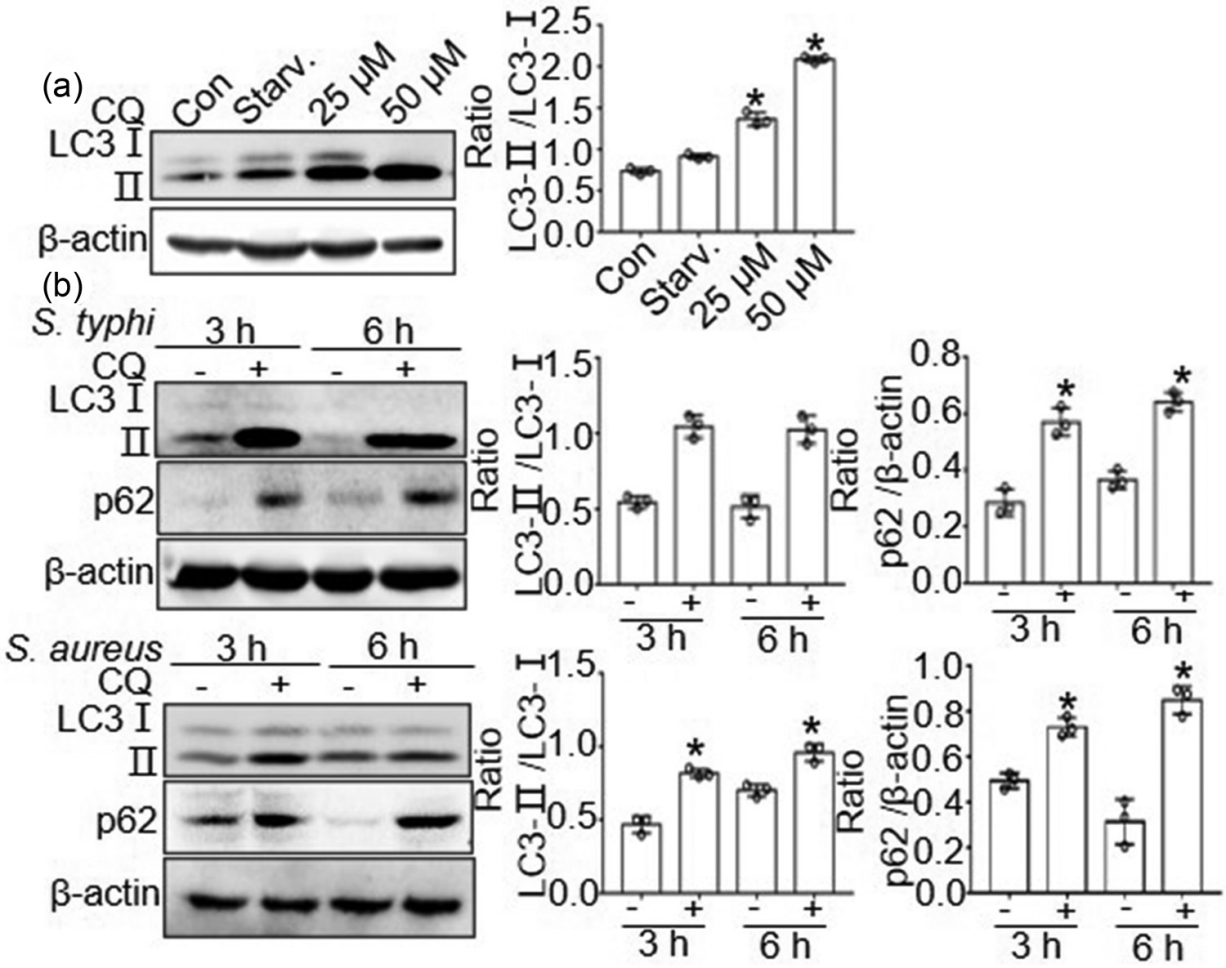
*S. typhimurium* and *S. aureus* accelerate autophagic flux. (a) The inhibitory efficiency of CQ, the inhibition of lysosome degradation. The protein expression of LC3 by CQ with difference concentration in BMDMs. Western blot was quantitatively analyzed, and data shown are mean ± SD from three independent experiments. *P < 0.05 compared with starvation. (b) The protein expression of LC3, p62 in CQ treated BMDMs at 3, 6 h after *S. typhimurium* or *S. aureus* infection. Western blot was quantitatively analyzed, and data shown are mean ± SD from three independent experiments.*P < 0.05 compared with non-treated BMDMs.

Autophagy flux is used to estimate autophagic activity and is defined as the amount of lysosome-dependent autophagy degradation. CQ, a potent V-ATPase inhibitor, is used to block lysosomal degradation and determine non-autophagic protein degradation to measure autophagic flux. As assessed by western blot, the protein expression of LC3-Ⅱ increased after treatment of BMDMs with 50 μM CQ, indicating that 50 μM was the most effective inhibitory concentration for further experiments (Figure 2a).

Next, we examined the effect of LC3 and p62 activation in *S. typhimurium-* and *S. aureus-*infected macrophages. The levels of LC3-Ⅱ and p62 protein increased upon CQ treatment during *S. typhimurium* and *S. aureus* infection (*P* < 0.05 versus non-treated with CQ) (Figure 2b), indicating that *S. typhimurium* and *S. aureus* infection accelerated autophagic flux.

### 3.3 BLP enhances autophagy formation in macrophages

**Figure 3:**
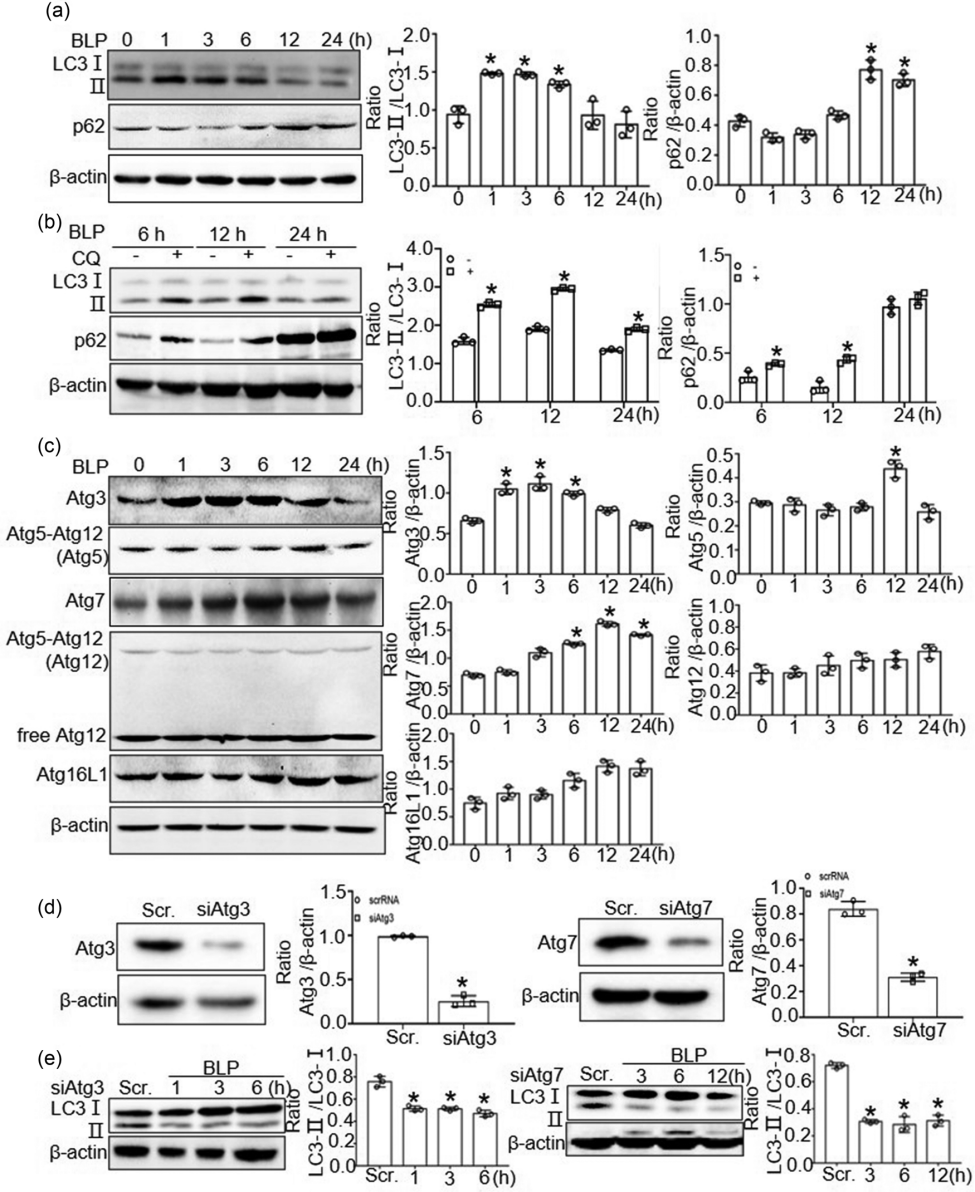
BLP induced autophagy in BMDMs. (a) The protein expression of LC3 and p62 in BMDMs at 0, 1, 3, 6, 12, and 24 h after BLP stimulation. Western blot was quantitatively analyzed, and data shown are mean ± SD from three independent experiments. *P < 0.05 compared with 0 h. (b) The protein expression of LC3 and p62 in CQ-treated BMDMs at 6, 12, and 24 h after BLP stimulation. Western blot was quantitatively analyzed, and data shown are mean ± SD from three independent experiments.*P < 0.05 compared with non-treated BMDMs. (c) The protein expression of autophagy associated protein in BMDMs after BLP stimulation. Western blot was quantitatively analyzed, and data shown are mean ± SD from three independent experiments. *P < 0.05 compared with 0 h. (d) BMDMs were transfected with Atg3 or Atg7-specific siRNA sequences or scrambled siRNA. The expression of Atg3 of Atg7 protein was assessed by western blot analysis. (e) BMDMs transfected with siAtg3 or siAtg7 were incubated with BLP and then the protein expression of LC3 was tested by Western blot. Western blot was quantitatively analyzed, and data shown are mean ± SD from three independent experiments. *P < 0.05 compared with scrRNA.

Next, we examined whether bacteria induced autophagy through BLP. We analyzed the expressions of autophagy-related proteins LC3 and p62 after BLP stimulation for 0, 1, 3, 6, 12, and 24 h in BMDMs. Enhanced protein expression of LC3-Ⅱ was observed at 1, 3, and 6 h and decreased at 12 and 24 h after BLP stimulation compared with levels at 0 h (*P* < 0.05); the protein expression trends of p62 were opposite to those of LC3-Ⅱ (Figure 3a). When we inhibited autophagy with CQ, the levels of LC3-Ⅱ and p62 protein increased upon CQ treatment after BLP stimulation (*P* < 0.05 versus non-treated with CQ) (Figure 3b). These findings indicate that BLP enhances autophagy formation in macrophages.

To investigate the mechanism of BLP-induced autophagy in BMDMs, we analyzed the expression of the five most common autophagy-related proteins: Atg3, Atg5, Atg7, Atg12, and Atg16L1. Enhanced protein expression of Atg3 was observed from 1 to 6 h and Atg7 was discovered at 6 and 12 h following BLP stimulation (*P* < 0.05 versus 0 h). However, the other factors did not show significant differences in the expression (Figure 3c). To confirm that Atg3 and Atg7 are involved in autophagosome formation, we transfected cells with Atg3 or Atg7 siRNA and confirmed effective Atg3 or Atg7 knockdown. The knockdown efficiency of siAtg3 and siAtg7 was nearly 74 and 63%, respectively. Western blot analysis showed that knockdown of Atg3 or Atg7 significantly attenuated BLP-enhanced protein expression of LC3-Ⅱ in BMDMs (Figure 3d and e).

### 3.4 BLP enhances apoptosis in macrophages

**Figure 4:**
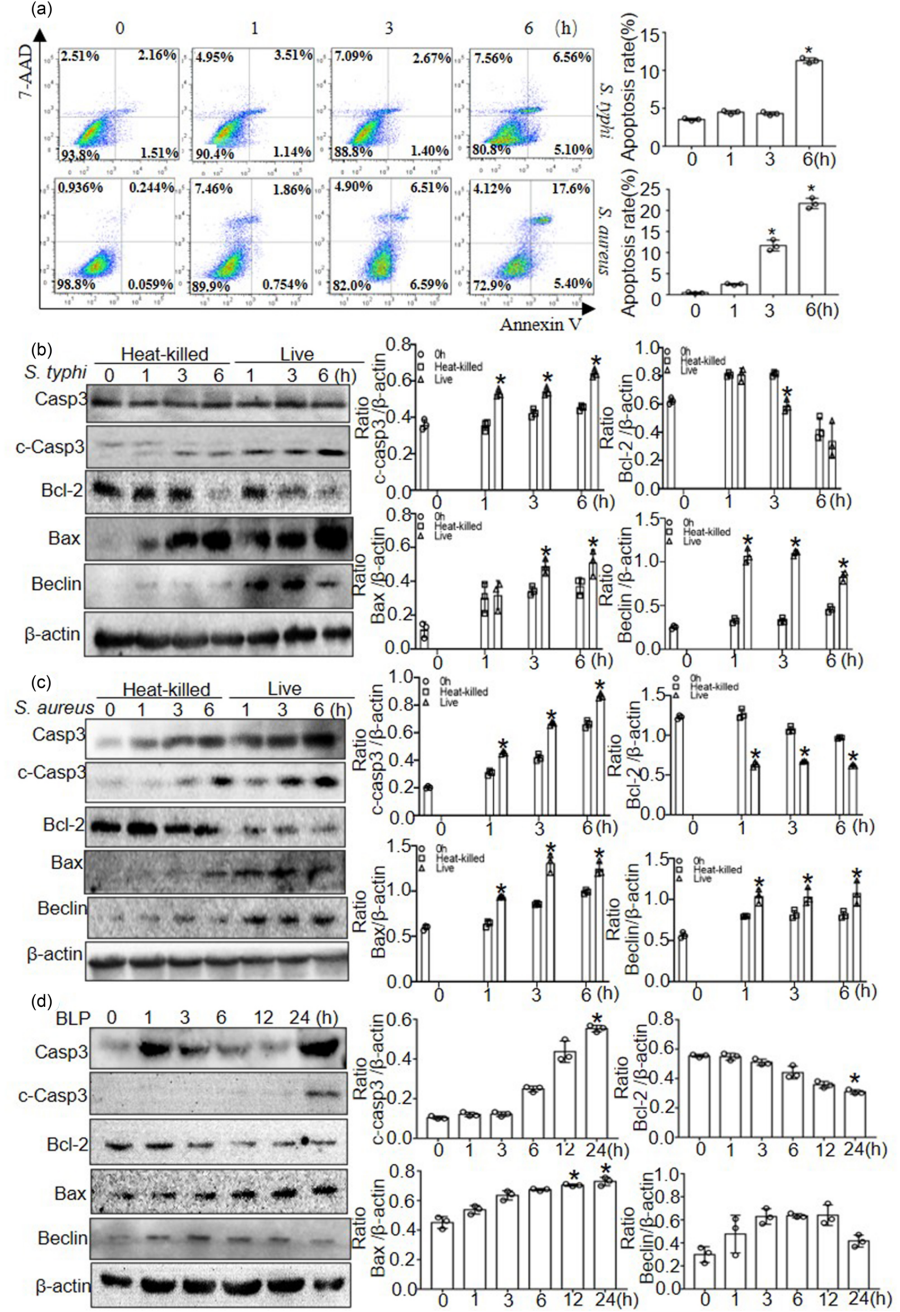
Apoptosis was induced by *S. typhimurium*, *S. aureus,* and BLP in macrophages. (a) The quantity of apoptotic cells determined by apoptosis assay kit used flow cytometric analysis. (b) and (c) The protein expression of caspase3, Bcl-2, Bax, and Becline-1 in BMDMs at 0, 1, 3, and 6 h after heat-killed and live *S. typhimurium* or *S. aureus* infection. Western blot was quantitatively analyzed, and data shown are mean ± SD from three independent experiments. *P < 0.05 compared with heat-killed bacteria. (d) The protein expression of caspase3, Bcl-2, Bax, and Becline-1 in BMDMs at 0, 1, 3, 6, 12, and 24 h after BLP stimulation. Western blot was quantitatively analyzed, and data shown are mean ± SD from three independent experiments. *P < 0.05 compared with 0 h.

We evaluated cell death using Annexin Ⅴ-FITC/PI double staining and flow cytometry to determine the apoptotic rate of macrophages after bacterial infection. As shown in Figure 4a, exposure to *S. typhimurium* and *S. aureus* significantly increased cell death in BMDMs compared with non-infected BMDMs. Next, we examined the protein expression of apoptosis-related proteins and found that proapoptotic-cleaved caspase-3, Bax, and Beclin expressions were increased after *S. typhimurium* and *S. aureus* infection at 3 and 6 h; in contrast, the protein expression of Bcl-2, an anti-apoptotic protein, was decreased upon bacterial infection. Moreover, heat-killed bacteria also induced apoptosis. The protein expression of cleaved-caspase3 and Bax were upregulated and Bcl-2 was downregulated; however, these effects were weaker than those observed with live bacteria (Figure 4b and c). Furthermore, we examined whether BLP stimulation induced apoptosis by western blot analysis. BLP stimulation slightly increased the expression of cleaved caspase-3 and Bax and decreased Bcl-2 expression at 24 h, and the expression of Beclin was not obviously changed (Figure 4d).

### 3.5 Inhibition of autophagy enhanced BLP-induced apoptosis

**Figure 5:**
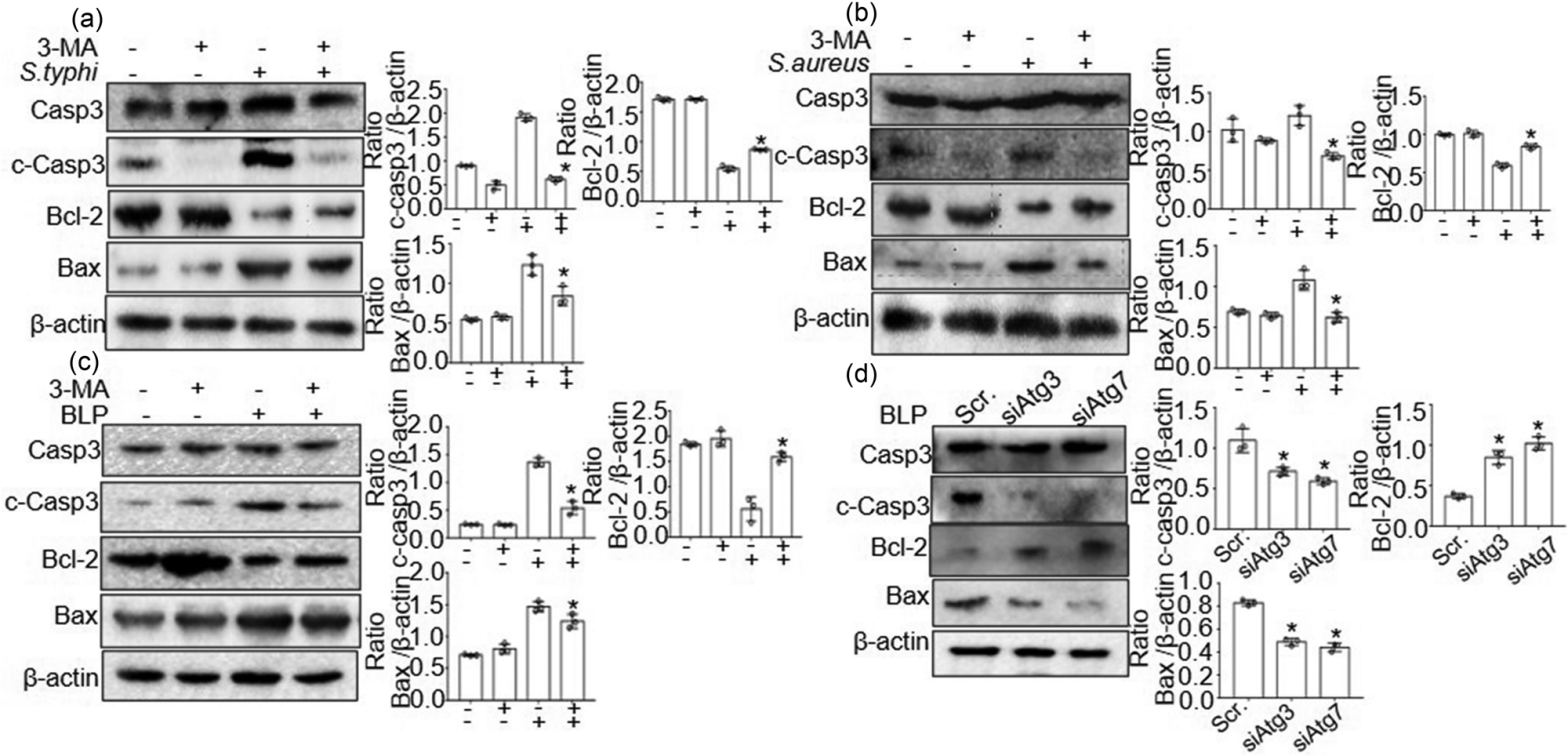
3-MA reduced apoptosis in BMDMs upon *S. typhimurium* and *S.aureus* infection and BLP stimulation. (a) and (b) The protein expression of caspase3, Bcl-2, and Bax in BMDMs at 3 h after *S. typhimurium* or *S. aureus* infection. Western blot was quantitatively analyzed, and data shown are mean ± SD from three independent experiments. *P < 0.05 compared with non-treated BMDMs. (c) The protein expression of caspase3, Bcl-2, and Bax in BMDMs at 24 h after BLP stimulation. Western blot was quantitatively analyzed, and data shown are mean ± SD from three independent experiments. *P < 0.05 compared with non-treated BMDMs. (d) BMDMs transfected with siAtg3 or siAtg7 and scrRNA after BLP stimulation for 24 h, and the protein expression of caspase3, Bcl-2, and Bax was assessed by Western blot. Western blot was quantitatively analyzed, and data shown are mean ± SD from three independent experiments. *P < 0.05 compared with scrRNA.

To study the correlation between bacterial infection-induced autophagy and apoptosis, autophagy was inhibited by 3-MA, and the expression of apoptosis-related proteins was examined. The levels of Bax and cleaved caspase-3 decreased, and Bcl-2 increased upon *S. typhimurium* and *S. aureus* infection at 3 and 6 h compared with levels in untreated conditions (Figure 5a and b). Furthermore, we investigated apoptosis after inhibiting autophagy by BLP challenge. The results were similar to bacteria stimulation by western blot analysis, which showed that the levels of Bax and cleaved caspase-3 decreased, and Bcl-2 increased with 3-MA treatment (Figure 5c). To further examine the relation between BLP-induced autophagy and apoptosis, we transfected BMDMs with siAtg3 or siAtg7. Western blot analysis showed that knockdown of Atg3 or Atg7 attenuated BLP-enhanced protein expression of c-caspase3 and Bax, and increased Bcl-2 expression in BMDMs (Figure 5d).

## 4 Discussion

Autophagy is important in both innate and adaptive immunity to bacterial infection. Bacterial infection triggers autophagy, which is initiated by conventional pattern recognition receptors (PRRs). A double-membrane compartment forms around the target bacteria and the cargo is transported to lysosomes for degradation. This process involves early TLR- and Nod-dependent detection of the released microbial products, such as LPS, DNA, peptidoglycan, and lipoprotein [13,14]. Previous research revealed that BLP is an abundant component in Gram-negative and -positive bacteria that induce the expression of pro-inflammatory cytokines. In Gram-negative bacteria, one-third of lipoprotein exists as a membrane-bound form with the peptidoglycan layer, and the function is about as potent as, if not more potent than LPS [7]. BLP, on the other hand, accounts for 2% or more of a Gram-positive bacterial proteome [15]. The deletion of BLP genes in *S. enterica serovar Typhimurium* decreased cytokine production, constrained bacterial load in various organs, and diminished organ damage in mice [7]. Thus, as a bacteria component, BLP may also be an important factor to induce autophagy. In the present study, both *S. typhimurium* and *S. aureus* induced autophagy, and heat-killed *S. typhimurium* could still induce autophagy. However, the activation of autophagy by heat-killed *S. aureus* was weak. Most of the studies demonstrated that *S. aureus* could induce autophagy. Maurer’s study discovered that autophagy could protect host cells against *S. aureus* infection by maintaining tolerance toward the pore-forming alpha-toxin secreted by *S. aureus* [16]. Agr-deficient *S. aureus* strains, the regulation gene of alpha-toxin, affect autophagosome formation after infecting cells [17,18]. Heat-killed *S. aureus* cannot induce autophagy because alpha-toxin can be eliminated after *S. aureus* is heated at 100°C for 30 min. Previous studies reported that heat-killed *S. typhimurium* effectively activated TLR2 and TLR4, and we speculated this was probably related to LPS, a component of the cellular wall of Gram-negative bacteria [19]. The interaction between LPS and TLR results in the activation of autophagy. *S. typhimurium* heated at 100°C for 30 min could not remove LPS, and hence, the autophagy was still activated. Moreover, because Gram-positive *S. aureus* lacked LPS and had its virulence destroyed by heat treatment, heat-killed *S. aureus* could not induce autophagy.

Autophagy, through a series of autophagy proteins, functions as a defense response against *S. aureus*. For example, Atg16L1 protects host cells by stimulating the release ADAM10 (a disintegrin and metalloproteinase 10) to scavenge bacterial toxins from *S. aureus,* and impaired Atg16L1 expression worsens *S. aureus-*induced mortality in mice. *S. aureus* can block autophagosome maturation via Atg5. These activities are related to the production of α-toxin by *S. aureus* [16,20,21]*. S. typhimurium* induces autophagy generally by ubiquitination. Ubiquitinated *Salmonella* is recognized by autophagy receptors and targeted to autophagosomes. Additionally, SopF produced by *S. typhimurium* type Ⅲ secretion systems (T3SS)-1 prevents Atg16L1 from being recruited by V-ATPase to damaged SCV membranes [22]. However, the mechanism of BLP-induced autophagy is unknown.

We found that BLP, the bacteria component, could activate autophagy independently. With bacteria, TLR2-dependent detection of the released BLP then activated autophagy via specific molecular cascades. Furthermore, we investigated autophagy-related proteins and found that BLP increased the protein levels of Atg3 and Atg7 to promote autophagosome formation. Atg7 can transfer Atg8 (LC3) to the E2-like enzyme Atg3 for transfer of Atg8 to the membrane lipid PE, and the resulting Atg8-PE can recruit cytoplasmic cargo to the isolation membrane for autophagosome incorporation [23]. Hence, the lack of Atg3 and Atg7 affects autophagosome formation. However, the study of Sharma et al. was different from ours, which demonstrated that the function of Atg5 played a crucial role in the activation of TLR2 signaling when embryonic fibroblasts were stimulated by Pam3CSK4 [24].

Autophagy and apoptosis are pathways through which macrophages maintain body homeostasis by killing pathogens and eliminating damaged cells via programmed cell death. Hence, the dynamic balance between autophagy and apoptosis during microbe infection plays a critical role in bacteria clearance. We found that apoptosis occurs at 1 h upon *S. typhimurium* and *S. aureus* infection and increases with the prolongation time through activating caspase-3. Similar to our study, another study found that *S. aureus* infection at 30, 60, and 90 min could significantly induce apoptosis of monocytes through caspase-3 activation. Heat-inactivated *S. aureus* nearly failed to induce apoptosis [25]. Hsin-Hung Lin’s study showed that *Salmonella* induced apoptosis of macrophages by activating caspase-3, -8, and -9 at 2 and 4 h [26]. In the early period of infection, *S. aureus* can induce cell apoptosis through virulence factors such as Staphylococcal enterotoxin and α-toxin [27]. *S. typhimurium* has evolved a myriad of mechanisms to counteract or exploit host responses through T3SS and T3SS effectors and has been found to interact with apoptotic cascades. Hence, many factors cause apoptosis by completing bacteria more quickly. However, we speculate that as a single bacterial component, BLP may take a longer or larger dose to induce apoptosis. Several other studies also suggested that Pam3CSK4 activated caspase-3 and increased apoptosis from 24 h [28,29]. As a commonly used PI3-kinase inhibitor, 3-MA is used to block the formation of autophagosomes and autophagic vacuoles [30]. Our results demonstrated that 3-MA effectively reduced the apoptosis of macrophages upon bacterial infection or BLP stimulation. A previous study indicated that excessive or uncontrolled autophagy activation induced by bacteria can lead to cell apoptosis. Furthermore, while transient autophagy may be protective, continuous autophagy activation results in inflammatory apoptosis [31]. Moreover, Chen’s results were also similar to ours. Statistically significant increases in Bcl-2 and decreases in both Bax and c-caspase-3 were observed after 3-MA intervention following LPS stimulation [32]. Furthermore, we found that apoptosis also decreased after the knockdown of Atg3 or Atg7 upon BLP stimulation. Some research indicated that a lack of Atg3 or Atg7 inhibits autophagy and further alleviates apoptosis [33]. For example, the expression of LC3-Ⅱ and c-caspase-3 was significantly reduced in Atg3 knockout cells after influenza A virus infection [34], and vancomycin-induced cell apoptosis was markedly decreased in Atg7-deficient cells [35]. Hence, inhibiting autophagy protects against apoptosis induced by bacteria and its components.

In conclusion, our study demonstrates that BLP as a bacteria component is an important factor in autophagy activation and induces macrophage apoptosis by *S. typhimurium* and *S. aureus*. Moreover, the knockdown of Atg3 and Atg7 or 3-MA inhibits BLP-induced macrophage apoptosis by suppressing autophagy activation. This study indicates another component common to both negative and positive bacteria that induces autophagy and apoptosis. Our results provide more information on the relation between autophagy and apoptosis.
